# Identification of inflammasome signaling proteins in neurons and microglia in early and intermediate stages of Alzheimer's disease

**DOI:** 10.1111/bpa.13142

**Published:** 2022-12-29

**Authors:** Regina T. Vontell, Juan Pablo de Rivero Vaccari, Xiaoyan Sun, Sakir Humayun Gultekin, Helen M. Bramlett, W. Dalton Dietrich, Robert W. Keane

**Affiliations:** ^1^ Department of Neurology University of Miami Brain Endowment Bank, University of Miami Miller School of Medicine Miami Florida USA; ^2^ Evelyn F. McKnight Brain Institute, Department of Neurology University of Miami Miller School of Medicine Miami Florida USA; ^3^ Department of Neurological Surgery and The Miami Project to Cure Paralysis University of Miami Miller School of Medicine Miami Florida USA; ^4^ Department of Physiology and Biophysics University of Miami Miller School of Medicine Miami Florida USA; ^5^ Center for Cognitive Neuroscience and Aging University of Miami Miller School of Medicine Miami Florida USA; ^6^ Department of Pathology University of Miami Miller School of Medicine Miami Florida USA; ^7^ Bruce W. Carter Department of Veterans Affairs Medical Center Miami Florida USA

**Keywords:** adaptor protein apoptosis‐associated speck‐like protein containing a caspase recruitment domain (ASC), Alzheimer's disease, caspase‐1, hyperphosphorylated tau, NOD‐like receptor proteins (NLRPs), β‐amyloid

## Abstract

Alzheimer's disease (AD) is a progressive neurodegenerative disease that destroys memory and cognitive function. Inflammasome activation has been suggested to play a critical role in the neuroinflammatory response in AD progression, but the cell‐type expression of inflammasome proteins in the brain has not been fully characterized. In this study, we used samples from the hippocampus formation, the subiculum, and the entorhinal cortex brain from 17 donors with low‐level AD pathology and 17 intermediate AD donors to assess the expression of inflammasome proteins. We performed analysis of hippocampal thickness, β‐amyloid plaques, and hyperphosphorylated tau to ascertain the cellular pathological changes that occur between low and intermediate AD pathology. Next, we determined changes in the cells that express the inflammasome sensor proteins NOD‐like receptor proteins (NLRP) 1 and 3, and caspase‐1. In addition, we stained section with IC100, a humanized monoclonal antibody directed against the inflammasome adaptor protein apoptosis‐associated speck‐like protein containing a caspase recruitment domain (ASC), and a commercially available anti‐ASC antibody. Our results indicate that hippocampal cortical thickness did not significantly change between low and intermediate AD pathology, but there was an increase in pTau and β‐amyloid clusters in intermediate AD cases. NLRP3 was identified mainly in microglial populations, whereas NLRP1 was seen in neuronal cytoplasmic regions. There was a significant increase of ASC in neurons labeled by IC100, whereas microglia in the hippocampus and subiculum were labeled with the commercial anti‐ASC antibody. Caspase‐1 was present in the parenchyma in the CA regions where amyloid and pTau were identified. Together, our results indicate increased inflammasome protein expression in the early pathological stages of AD, that IC100 identifies neurons in early stages of AD and that ASC expression correlates with Aβ and pTau in postmortem AD brains.

## 1 INTRODUCTION

Alzheimer's disease (AD) is characterized clinically by functional and cognitive impairment, particularly memory loss [1, 2]. The pathomechanisms that contribute to AD include the accumulation of misfolded protein aggregates of extracellular amyloid‐β (Aβ), intracellular hyperphosphorylated tau (pTau) neurofibrillary tangles, and chronic neuroinflammation [1, 2]. Anatomically, the hippocampus is affected early in the disease and then the neurodegeneration progresses throughout the cerebral cortex [1, 2].

Emerging evidence supports the idea that there is a link between the misfolded protein aggregates and the activation of the inflammasome of the innate immune system [3]. The inflammasome is comprised of caspase‐1, apoptosis‐associated speck‐like protein containing a caspase recruitment domain (ASC), and a sensor protein such as NOD‐like receptor1 (NLRP1) or NLRP3 [4]. The sensor NLR protein binds to pathogen‐associated molecular patterns or danger‐associated molecular patterns to activate the inflammasome [5]. Inflammasome activation is accompanied by oligomerization of the sensor that then recruits monomeric ASC which oligomerizes with an NOD‐like receptor such as NLRP3 via homotypic interactions between the PYRIN domain (PYD) of ASC and the PYD of NLRP3 [6, 7]. Upon aggregation of ASC with NLRP3, pro‐caspase‐1 is recruited and binds to ASC via homotypic CARD‐CARD interactions between the two proteins. The activated inflammasome leads to the cleavage of caspase‐1 into its active form, leading to the production of mature interleukin (IL)‐1β and IL‐18 [8]. These pro‐inflammatory cytokines are then secreted to spread the inflammatory signal. Moreover, active caspase‐1 also cleaves gasdermin‐D (GSDM‐D) [9]. Upon GSDM‐D cleavage, the N‐terminus is inserted into the cell membrane to form a pore through which IL‐1β and IL‐18 are released, resulting in pyroptosis [9]. Pyroptosis leads to inflammasome proteins being released into the extracellular space, including the release of oligomerized ASC in the form of ASC specks that present prion‐like properties [10, 11]. Extracellular ASC specks remain present for long periods of time, while retaining their ability to cleave pro‐IL‐1β, and thus, perpetuating inflammation [12]. Importantly, this prion‐like propagation of inflammation by ASC specks contributes to a progressive inflammatory state that plays a central role in neurodegeneration [13].

Recent studies have implicated an important role for the NLRP3 inflammasome and ASC specks in the spread of the inflammatory response and the aggregation of misfolded proteins characteristics of neurodegenerative diseases [3]. For instance, Aβ aggregates trigger NLRP‐3 inflammasome activation in microglia [14]. Importantly, extracellular ASC specks directly cross‐seed Aβ aggregates in vitro and in vivo [11]. Moreover, therapeutics targeting the NLRP3 inflammasome [15, 16] and ASC specks [11] have shown promising results in animal models of AD [17].

Previous studies using anti‐ASC antibodies have shown that this therapeutic approach reduces pathology in several indications, including spinal cord injury [8], traumatic brain injury [18], acute lung injury [19, 20, 21], multiple sclerosis [22] and aging [23]. We have developed IC 100, a humanized and deimmunized monoclonal antibody (mAb) (IgG4k) against ASC [22], and in this study, we used IC100 and a commercially available anti‐ASC antibody to determine the cell‐type distribution of ASC in postmortem brains from donors with AD. Moreover, we also employed a panel of commercially available antibodies to identify Aβ and pTau, and the inflammasome proteins, NLRP‐1, NLRP‐3, and caspase‐1 in postmortem human brains with and without intermediate AD neuropathological changes.

## 2 MATERIALS AND METHODS

### 2.1 Postmortem human brains

Informed consent was acquired for postmortem examination research according to the University of Miami, Institutional Regulatory Board (IRB) guidelines.

### 2.2 Tissue preparation

Seventeen postmortem brains from donors with intermediate AD (intermediate AD neuropathological changes (Braak III–VI) average age 83.41 ± 7.05 years; 10 females and 7 males) were used in this study, and 17 age‐matched non‐demented controls with age‐related neuropathological changes (low AD neuropathological changes (Braak 0–II); average age 84.59 ± 10.26 years; 10 females and 7 males). The neuropathological staging was based on a complete histological assessment including samples taken from 20 different brain regions and a panel of 15 immunohistochemical stains (anti‐mouse monoclonal AT‐8 (ptau) immunostaining completed in 7 brain regions and anti‐mouse monoclonal beta‐amyloid immunostaining completed in 8 brain regions) and 7 brain regions stained with Bielschowsky Silver Technique. The diagnosis of cognitive status was provided by a clinician as noted in the patient charts (i.e., based on clinical assessment or MMSE score) or ascertained via the Modified Telephone Interview for Cognitive Status (TICS‐M) before death for [24, 25] and re‐evaluated by a cognitive neurologist (X.S.). The TICS‐M score was analyzed using the optimal cutoff scores described in [26]. In brief, using the TICS‐M scores that were in the 62% range (e.g., ≥31/50) were characterized as no cognitive disorders [26]. Dementia was described as either moderate dementia or severe dementia and was determined by the clinical notation and reviewed by the cognitive neurologist. Cases with clinical findings of non‐AD neurodegenerative diseases were excluded from the study. The primary cause of death in each case was given by the clinician and the level of AD neuropathological changes were confirmed by a neuropathologist (S.H.G.). The final diagnosis of low AD neuropathological changes without dementia or intermediate AD neuropathological changes with dementia was established based on clinical assessment and neuropathological findings at consensus review meeting. Clinical details can be found in Table 1.

**TABLE 1:** Summary of the clinical details of the human postmortem cases

Age	Sex	PMI	A	C	B	Braak	Clinical assessment	COD	Race
Low AD									
188	F	9.00	1	1	1	Stage I	No Cognitive Disorder	Arteriovascular sclerotic disease	Caucasian
287	M	8.00	1	0	1	Stage I	No Cognitive Disorder	Cardiac Arrest; Atherosclerotic Disease	Caucasian
386	F	37.00	1	0	1	Stage II	No Cognitive Disorder	urethral cancer, heart murmur	Caucasian
489	F	4.55	1	1	1	Stages I–II	No Cognitive Disorder	Coronary Artery Disease; Atrial fibrillation; Hypertension	Caucasian
596	F	6.00	0	1	1	Stages I–II	No Cognitive Disorder	Generalized atherosclerosis	Caucasian
691	F	18.66	0	0	1	Stage I	No Cognitive Disorder	End Stage Congestive Heart Failure	Caucasian
792	F	22.30	1	0	1	Stage II	No Cognitive Disorder	Atherosclerotic Heart Disease	Caucasian
872	M	17.25	0	0	1	Stage I	No Cognitive Disorder	Atherosclerotic Coronary Artery Disease, Ischemic Cardiomyopathy	Caucasian
972	M	17.66	0	0	0	Stage 0	No Cognitive Disorder	Acute Myocardial Infarction; Diabetes Mellitus	Caucasian
1073	M	20.50	2	0	1	Stage I	No Cognitive Disorder	Atherosclerotic Heart Disease; Cardiomyopathy; Diabetes	Caucasian
1174	F	34.9	1	0	1	Stage I	No Cognitive Disorder	chronic kidney disease	Caucasian
1267	F	25.95	1	0	1	Stage 1	No Cognitive Disorder	Atherosclerotic Coronary Artery Disease, Tobacco Abuse, Hypertension	Caucasian
1394	M	23.40	2	0	1	Stage 1	No Cognitive Disorder	Congestive Heart Failure	Caucasian
1490	F	24.30	1	1	1	Stage II	No Cognitive Disorder	Cardiac Arrest; Respiratory Arrest; Unspecified Natural	Caucasian
								Causes; Cardiovascular Disease	
1593	M	16.88	1	1	1	Stages I–II	No Cognitive Disorder	Cardiac Arrest; Atherosclerotic Disease	Caucasian
1672	M	24.28	1	0	0	Stage 0	No Cognitive Disorder	Acute Myocardial Infarction caused by Hypertensive	Caucasian
								Arteriosclerotic Cardiovascular Disease	
17102	F	10.30	0	0	1	Stage II	No Cognitive Disorder	Failure to thrive	Caucasian
Mean 84.588		18.88							
Intermediate AD									
192	M	12.00	3	3	2	Stages III–IV	Moderate	Acute Renal Failure	Caucasian
290	F	8.48	2	3	2	Stage III	Moderate	Acute Renal Failure	Caucasian
390	F	22.12	2	3	2	Stages III–IV	Severe	Advanced Dementia; vascular	Caucasian
491	F	15.00	3	2	2	Stage III	Moderate	Lung cancer	Caucasian
579	F	18.28	2	3	2	Stage III	Moderate	Cardiac Arrest; Atherosclerotic Disease	Caucasian
682	M	17.60	2	3	2	Stages III–IV	Moderate	Cardiac Arrest; Atherosclerotic Disease	Caucasian
794	F	18.45	2	2	2	Stages III–IV	Moderate	Cardiopulmonary Arrest	Caucasian
882	M	18.22	2	2	2	Stages III–IV	Moderate	Cardiac Arrest; Vascular Dementia	Caucasian
987	M	13.91	2	3	3	Stages V–VI	Moderate	Sepsis caused by urinary tract infection	Caucasian
1080	M	10.53	3	2	3	Stages V–VI	Moderate	Cardiac Arrest; Atherosclerotic Disease	Caucasian
1178	M	14.51	2	2	2	Stages III–IV	Moderate	Cardiac Arrest; Atherosclerotic Disease	Caucasian
1278	F	6.00	2	2	2	Stages III–IV	Moderate	Cardiac Arrest; Atherosclerotic Disease	Caucasian
1379	F	6.40	1	3	3	Stages V–VI	Severe	Cardiac Arrest; Atherosclerotic Disease	Caucasian
1466	F	44.25	3	3	2	Stages III–IV	Moderate	Urinary Tract Infection	Caucasian
1583	M	15.12	2	2	2	Stages III–IV	Moderate	Liver Failure	Caucasian
1677	F	10.75	2	2	2	Stage III	Moderate	Cardiac Arrest; Atherosclerotic Disease	Caucasian
1790	F	13.25	2	1	2	Stages III–IV	Moderate	Lung Cancer	Caucasian
Mean 83.412		15.58							

*Note*: PMI = postmortem interval; Yr = year; A Score = Aβ immunopositivity, Thal Phase; C Score = neuritic plaque density, CERAD; B Score = Neurofibrillary tangles; COD = Cause of Death; AD = Alzheimer's disease; M = male; F = female; No Cognitive Disorder ≥62% on Modified Telephone Interview for Cognitive Status (TICS‐M; score 61–54%); Moderate = Moderate dementia; Severe = Severe dementia.

In brief, whole brains were procured on a postmortem interval (PMI) of an average of 17.23 ± 8.72 h after the time of death. The left hemisphere was frozen, and the right hemisphere was fixed in 10% formalin (pH 7.0) for 1 month, and then it was sliced, sampled and embedded in paraffin blocks that were processed on a Leica Tissue‐Tek Processor (Leica Biosystems). Paraffin‐embedded tissue sections were then taken from the hippocampus formation, which included the entorhinal cortex, the Cornu Ammonis (CA) regions CA1–CA3, the subiculum, and the dentate gyrus (DG) and were used for immunohistochemical and histochemical staining as described below. Paraffin‐embedded tissue‐blocks were sectioned at a 20‐μm thickness with 20 retrieved serial sections using a Leica RM2245 microtome (Leica Microsystems Ltd.). Three sections based on a systematic sampling principle and a section‐sampling fraction of 1/5 [27] were selected from each block for further investigations.

### 2.3 Immunohistochemistry and histochemistry

Standard immunohistochemistry procedures for 20‐μm‐thick brain sections have been described previously in [28, 29]. In brief, after deparaffinization, endogenous peroxidase activity was quenched by placing the slides into 3% hydrogen peroxide (H_2_O_2_) for 10 min. Sections were immersed in preheated 10 mM citric acid (VWR), pH 6.0, for 30 min and cooled in cold water or pretreated with formic acid (Sigma‐Aldrich) for 5 min. Sections were then blocked in 5% goat serum (Vector Laboratories) for 20 min before being incubated overnight at 4°C in a solution of mouse anti‐Phospho‐Tau (Ser202, Thr205) Antibody (AT‐8; 0.4 μg/ml; Thermo Fisher Chemicals), mouse anti‐β‐Amyloid (6E10; 1.0 μg/ml; BioLegend), rabbit anti‐NLRP3 (3.0 μg/ml; MilliporeSigma), mouse anti‐NLRP1 (1.0 μg/ml; Enzo Life Sciences), mouse anti‐ASC (B‐3; Santa Cruz Biotechnology, 0.4 μg/ml) human anti‐ASC (IC100, 2 μg/ml; as described in [22, 23]) and rabbit anti‐caspase 1 (0.6 μg/ml; MilliporeSigma) in PBS. The next day, sections were exposed to biotinylated horse‐anti‐mouse IgG or biotinylated goat‐anti‐rabbit IgG secondary antibody (15 μg/ml; Vector Laboratories) in PBS for 1 h followed by avidin‐biotin complex for 1 h (1:200, ABC; Vector Laboratories). Reactions were visualized with 3,3′‐diaminobenzidine (MilliporeSigma) for 10 min. Finally, the sections were dehydrated, cleared in xylene, and cover slipped. As negative low AD, we performed staining in the absence of the primary antibodies and no specific staining was identified in these preparations. In addition, we used a sample of skin and tonsil as positive low AD and compared the immunoreactivity described in [30] and https://www.proteinatlas.org/ (Figure S1).

### 2.4 Immunofluorescence labeling

To identify the cellular location of the NLRP proteins, immunofluorescence (IF) triple‐labeling was performed on the intermediate AD and on the low AD samples. Primary antibodies rabbit anti‐NLRP3 (3.0 μg/ml) and mouse anti‐NLRP1 (1.0 μg/ml) were mixed in separate cocktail solutions with compatible antibodies, as follows: chicken polyclonal anti‐ionized calcium‐binding adaptor molecule 1 (Iba‐1; 0.1 μg/ml) or rabbit polyclonal Iba‐1 (0.5 μg/ml; Wako Chemicals) as a marker for microglia and macrophages and guinea pig neuronal nuclear antigen (NeuN; 0.3 μg/ml; MilliporeSigma) and a marker for neurons. Sections were pretreated as described above and blocked in 5% goat serum for 20 min before the primary antibodies were applied and incubated overnight at 4°C. Following primary antibody incubation, sections were rinsed three times in PBS, for 3 min before the secondary antibodies were added. The samples were finally soaked for 1.5 h in PBS containing secondary antibody cocktail: goat anti‐mouse IgG conjugated to Alexa Fluor 488 (4 μg/ml; Invitrogen) or goat anti‐rabbit IgG conjugated to Alexa Fluor 488 (4 μg/ml; Invitrogen). Included in the cocktails were goat anti‐chicken IgG conjugated to Alexa Fluor 546 (4 μg/ml; Invitrogen) or goat anti‐rabbit IgG conjugated to Alexa Fluor 546 (4 μg/ml; Invitrogen) and goat anti‐guinea pig conjugated to Alexa Fluor 647 (4 μg/ml; Invitrogen). Finally, the sections cover slipped using ProLong Gold antifade reagent (Invitrogen) and kept in the dark at 4°C until analysis.

### 2.5 Microscopic analyses

**FIGURE 1:**
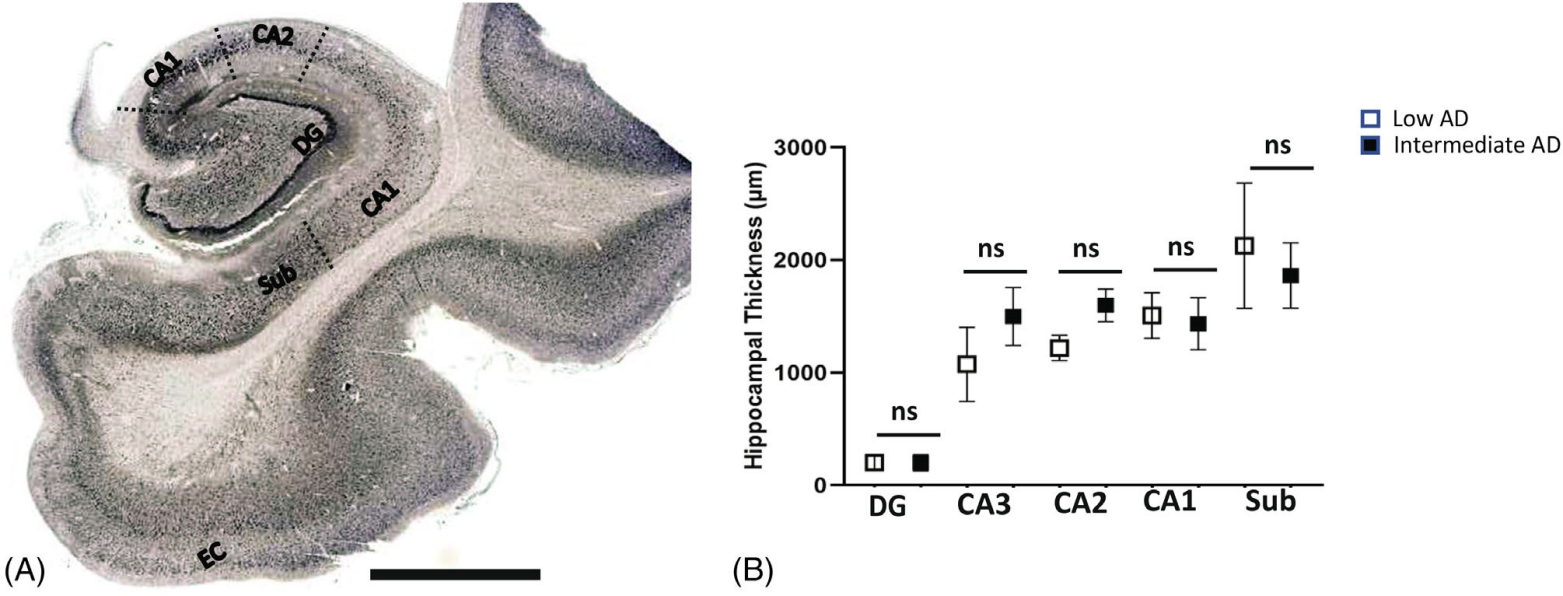
Global view of the hippocampus. Strategy of the quantification of Aβ, ptau, and the inflammasome components is shown in the diagram. (A) Dotted lines represent the boundaries that were made in hippocampal regions of the dentate gyrus (DG); Cornu Ammonis Field 3 (CA3); Cornu Ammonis Field 2 (CA2); Cornu Ammonis Field 1 (CA1); and the subiculum (sub). (B) Comparison of hippocampal formation thickness between low AD and intermediate AD. Scale = 1 mm; ns = not significant; micron = μm.

Hematoxylin and eosin (H&E) staining was used to evaluate the general morphology of the brain tissue and orientation of the brain regions as described previously [31]. For H&E staining, standard tissue paraffin block was sectioned at 20‐μm thickness and the slides were allowed to dry and heated at 60°C for 30 min. Before staining, sections were deparaffinized in three changes of xylene and rehydrated through graded concentrations of ethanol. Sampling areas and the strategies for identifying regions of interest were shown using standard H&E‐stained sections (Figure 1A).

Multichannel confocal triple‐labeled microphotographs were captured using a Leica SP8 spectral confocal microscope with settings appropriate for the fluorophore. Images were captured with a Leica microscope DM6000 B using a 40× objective (Leica Microsystems Ltd.) and processed in Adobe Photoshop (version 11.0.2; Adobe Systems Inc.).

### 2.6 Hippocampal thickness

Images of H&E‐stained sections were obtained using the standard virtual tissue scan (EasyScan, Motic Microscopes) at a 40× magnification as described in [32]. Unbiased measurement of thickness of the hippocampus was done using the “Incremental Distances” plugin (Image‐Pro Premier; Media Cybernetics) to measure the distance between the white matter boundary and the edge of the hippocampus formation (i.e., edge of stratum *oriens* to the edge of the stratum *lacunosum*) using perpendicular lines with a 10‐μm step to give an average of 100 measures, in an average area of 3 mm^2^ per slide.

### 2.7 Neurofibrillary tangles, neuritic plaques, and ASC positive counts

Unbiased cell counts of AT‐8 pTau positive neurons, ASC positive cells, and Aβ clusters in 1 mm^2^ of tissue were obtained using the extended Depth of Field (EDF) virtual tissue scan, which allowed for a series of *Z*‐stack images to be transformed into a single image (EasyScan, Motic Microscopes). Three contours from each region of interest (ROI) were taken from the CA1, CA2, CA3, the subiculum, and the adjacent DG, which encompassed an average area of 3.2 mm^2^ per region using the Image‐Pro Premier (Media Cybernetics) program. ROIs were determined by the cellular architecture as described in [33] and sampled from the postmortem brains: CA1 was sampled from regions anterior to the DG, CA2 was sampled from the region posterior to the DG and CA3 was sampled from regions that are adjacent to the opening of the DG. For each region, the CA scans encompassed all layers of the strata.

Cellular densities of positive‐stained cells and Aβ clusters in all contours were quantified by investigators who were blinded to case data. Tissue scans were reviewed (by RV) to ensure that counts had met the criteria to avoid duplication of counts (e.g., an area containing a positive nucleus [>10 μm^2^] connected positive processes or Aβ clusters [>20 μm^2^]). In a pilot study, we confirmed that the counting profile described previously counted the correct number of labeled cells and nuclei (using an ImageJ cell counter).

Estimation of number density was performed by applying the following formula [34]:

$$N=\frac{\sum Q^{-}}{V}$$

where *N* is the total number of cells or clusters per volume of brain region; ∑*Q* ^−^ is the number of counted cells; and *V* is the volume of regions of interest per sampling frame.

### 2.8 Data analysis

In this study, we used a Student's t test to compare the variables between two groups (AD and control) in all experiments. Data were presented as mean ± standard deviation (SD); significance was assumed at *p* < 0.05. All statistical analyses and generation of plots were performed using GraphPad Prism 9.0 (GraphPad Software).

## 3 RESULTS

### 3.1 Donors and AD pathology

Experiments in this study were designed to determine the density and distribution of Aβ and pTau, and the expression of inflammatory proteins, NLRP1, NLRP3, ASC, and caspase‐1 in postmortem human brains with and without intermediate AD neuropathological changes. Neuropathological scores and demographics are shown in Table 1.

The Braak score in the low AD group ranged from 0 to II; none to low AD neuropathological change. The neuropathological score for the distribution of neurofibrillary tangles ranged from B0 to B1 with a mode of B1 (i.e., neurofibrillary tangles were mainly seen in the subiculum and CA1). The Aβ diffuse plaque distribution score, Thal phase, and A score, ranged from A0 to A2, with a mode of A1 and the neuritic plaque density or CERAD score, ranged from C0 to C1 with a mode of C0. Causes of death were caused by the complications of cardiovascular disease (14 donors), renal failure (1 donor), cancer (1 donor), and liver failure (1 donor).

Donors with intermediate AD had Braak Scores that ranged from III to VI; intermediate neuropathological change. The neuropathological score for the distribution of neurofibrillary tangles ranged from B2 to B3 with a mode of B2 (i.e., neurofibrillary tangles were seen throughout the subiculum and CA1–CA3). The Aβ diffuse plaque distribution score, Thal phase, and A score, ranged from A1 to A3, with a mode of A2 and the neuritic plaque density or CERAD score, ranged from C1 to C3 with a mode of C2. The intermediate AD group had similar complications as the low AD group such as cardiovascular disease (10 donors), renal failure (4 donors), cancer (1 donor), and liver failure (1 donor). Ages of the low AD donors were not significantly higher than that of donors with intermediate AD (*p* > 0.05). Additionally, the two groups were not significantly different with respect to PMI time (*p* > 0.05).

### 3.2 Hippocampal assessment

Any changes in cellular density in a CA region could result from shrinkage in the strata caused by the neuropil changes [35, 36]. Moreover, a decrease in strata volume may be related to a difference in layer thickness [37]. To address this possibility, the hippocampal strata were measured between the gray/white matter interface and the alveus surface. There was no significant difference in hippocampal thickness between intermediate AD cases and low AD (Figure 1B) when the hippocampal thickness was measured in the CA1 (*p* = 0.7; Intermediate AD 1432 ± 232, *n* = 17; Low AD 1508 ± 203, *n* = 17), CA2 (*p* = 0.1; Intermediate AD 1598 ± 145, *n* = 17; Low AD 1219 ± 112, *n* = 17), CA3 (*p* = 0.4; Intermediate AD 1499 ± 256, *n* = 17; Low AD 1074 ± 329, *n* = 17) regions, and subiculum (*p* > 0.97; Intermediate AD 1863 ± 290, *n* = 17; Low AD 2127 ± 556, *n* = 17), indicating that changes in cellular populations are not related to changes in hippocampal volume.

### 3.3 The number of Aβ clusters does not differ between intermediate AD and low AD in the hippocampus

**FIGURE 2:**
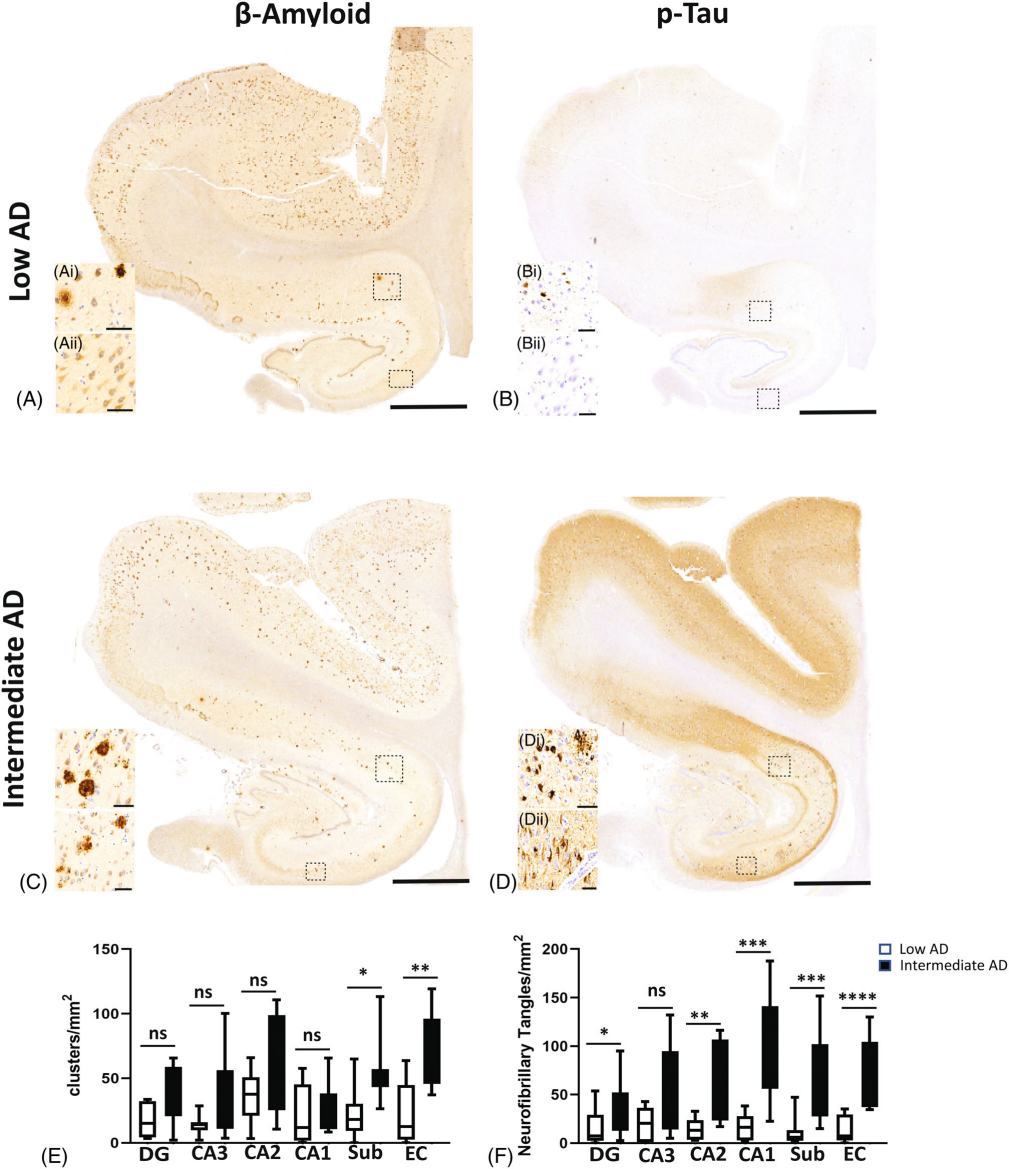
AD neuropathological changes seen with Aβ and pTau. Aβ (A) and ptau (B) in cases with Braak 0‐II and low AD neuropathological changes (low AD). AD neuropathological changes seen in intermediate AD with Braak scores of III–VI (C: Aβ and D: pTau). (E) Distribution of diffuse and neuritic Aβ plaques in intermediate AD cases compared with control. (F) Number of neurofibrillary tangles in hippocampal regions of the DG, CA1, CA2, CA3, and subiculum regions and in the entorhinal cortex between intermediate AD cases compared with low AD. Alzheimer's disease (AD), beta‐amyloid (Aβ), hyperphosphorylated (ptau), dentate gyrus (DG); Cornu Ammonis Field 3 (CA3); Cornu Ammonis Field 2 (CA2); Cornu Ammonis Field 1 (CA1); subiculum (sub); entorhinal cortex (EC); ns = not significant; * = *p* < 0.05; **** = *p* < 0.0001; scale bar = 60 μm.

Pathologic change that incorporates histopathologic assessments of Aβ deposits and the staging of neurofibrillary tangles [38]. It is important to determine if the amount of Aβ clusters alters between low AD and intermediate AD in our ROIs. In all of the CA regions and in the DG of the hippocampus, the number of Aβ clusters did not present a significant change in the intermediate AD cases compared to low AD (*p* > 0.05; Figure 2A,C,E). However, there was a significant increase in Aβ deposits found in the intermediate AD cases compared to the low AD cases, in the subiculum (*p* = 0.02; Intermediate AD 54.08 ± 25.62, *n* = 17; low AD 22.94 ± 20.75, *n* = 17) and in the entorhinal cortex (*p* = 0.01; Intermediate AD 71.27 ± 28.43, *n* = 17; low AD 23.62 ± 24.46, *n* = 17). Outside of the ROIs, we investigated the neuropathological scores of diffuse Aβ plaques (e.g., the Thal score or A score) identified in the case assessments. The intermediate AD cases had an average Thal score of “A2” and an average CERAD (e.g., the extent of neuritic amyloid plaques or C score) score of “C2,” whereas in the low AD group, the average Thal score was “A1” and the average CERAD was “C0.” The increase of Aβ plaques in the subiculum and the entorhinal cortex demonstrates that there are numerous regions that are prone to have a significant change in protein accumulation as the neural degeneration progresses.

### 3.4 Tau positivity increased in CA1 in intermediate AD cases

The levels of pTau are used to assign Braak staging based on the occurrence of neurofibrillary tangles and neuritic threads [39]. We sought to find if the number of neurons with neurofibrillary tangles alters between intermediate AD and low AD. A significant increase in the number of pTau positive neurons was seen in the intermediate cases compared to the low AD cases in the subiculum (*p* = 0.0006; Intermediate AD 64 ± 48, *n* = 17; low AD 6.2 ± 14, *n* = 17) and entorhinal cortex (*p* < 0.0001; Intermediate AD 55 ± 36, *n* = 17; low AD 7.5 ± 14, *n* = 17). In the hippocampus, there was a significant increase in pTau in the intermediate AD group in the DG (*p* < 0.04; Intermediate AD 39 ± 27, *n* = 17; low AD 7.5 ± 2.8, *n* = 17), the CA2 (*p* < 0.002; Intermediate AD 99 ± 56, *n* = 17; low AD, 16 ± 8, *n* = 17) and the CA1 region (*p* < 0.0002; Intermediate AD 99.96 ± 57, *n* = 17; low AD 16.33 ± 13, *n* = 17), but not in the CA3 (*p* > 0.05; Figure 2B,D,F). The increase in neurofibrillary counts exemplifies the extent of neurodegeneration occurring in specific hippocampal regions. These data also show that certain regions of the brain (e.g., CA3) do not alter in the number of neurofibrillary tangles, thus there are specific areas spared at this stage of AD pathology.

### 3.5 NLRP3 is mainly present in microglia‐like structures

**FIGURE 3:**
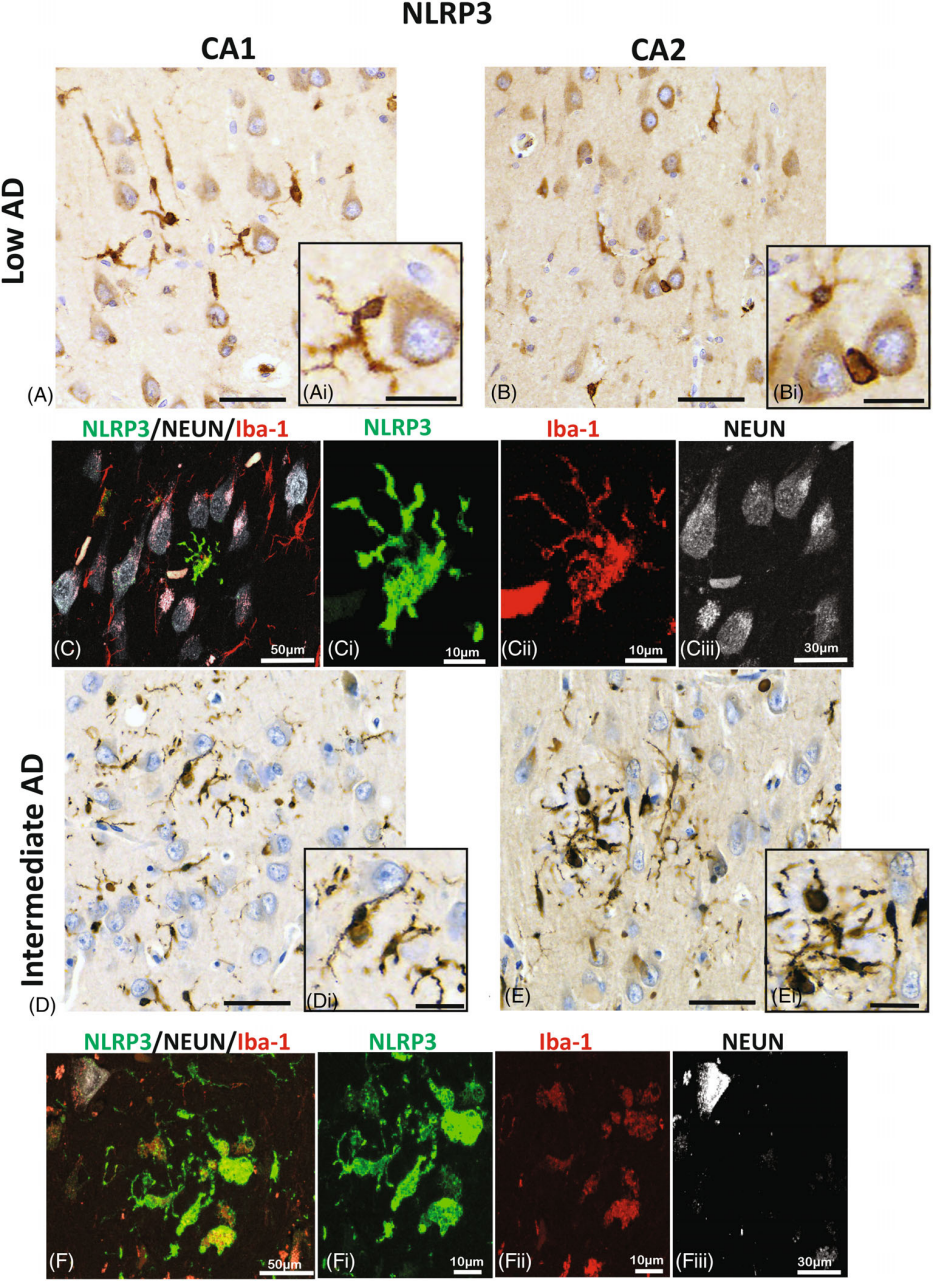
Localization of NLRP3 in microglia. Immunoreactivity of NLRP3 in the CA1 (A and D) and CA2 (B and E) hippocampal regions in a representative section of low (A–C) and in an intermediate AD (D–F) case. Expression of NLRP3 is seen mainly in the processes of the ramified microglia of low AD (A, Ai and B, Bi). The expression of NLPR3 (green) is confirmed by the confocal photomicrographs, imaged in the CA2 hippocampal region (C), where there is expression in the microglia (red; Cii) and not in the neurons (white; Ciii). Whereas, in the intermediate AD the expression densely stains the processes of activated amoeboid‐like microglia (D, Di and E, Ei). The prominent expression of NRLP3, is demonstrated in the CA2 hippocampal region (green, F and Fi) in the microglia cells (red, Fii), but absent in the neurons (Fiii). Alzheimer's disease (AD); NOD‐like receptor proteins (NLRP); Cornu Ammonis Field 2 (CA2); Cornu Ammonis Field 1 (CA1); images A, B, D, and E; scale bars = 60 μm; insert scale bars = 30 μm.

In low stages of AD, tau proteins accumulate in the entorhinal cortex, CA1, and the subiculum hippocampal regions [40]. As AD progresses, the pathology advances to the CA2, CA3, and DG hippocampal regions with an increase in neurofibrillary tangles and amyloid depositions [40, 41]. Recent understanding of the pathophysiological of AD suggests that the CA1/CA2 border zone is imperative for social recognition and memory processing [42]. While increased pTau positive neurons in the CA2 region seen in postmortem cases of intermediate AD have been well reported [38, 39, 40], the distribution and location of the inflammasome formation in AD cases have not been well characterized. Hence, we focused on the CA1/CA2 border zone to investigate the senor NLR3; here, we found that in the low AD, NLRP3 was present in all regions of the hippocampus with equal distribution patterns (CA1: Figure 3A and CA2: Figure 3B). Immunofluorescence labeling identified the expression of NLRP3 in microglia and it was not observed in the neuronal population (CA 2; Figure 3C). Similarly, in the intermediate AD cases, we show whorls of NLRP‐3 positive microglia in both, the CA1 (Figure 3D) and CA2 (Figure 3E) regions of the hippocampus, indicating that NLRP3 is present in microglia (CA2; Figure 3F) in two different regions of the hippocampus, namely CA1 and CA2.

### 3.6 Perinuclear expression of NLRP1 is present in hippocampal neurons of CA1

**FIGURE 4:**
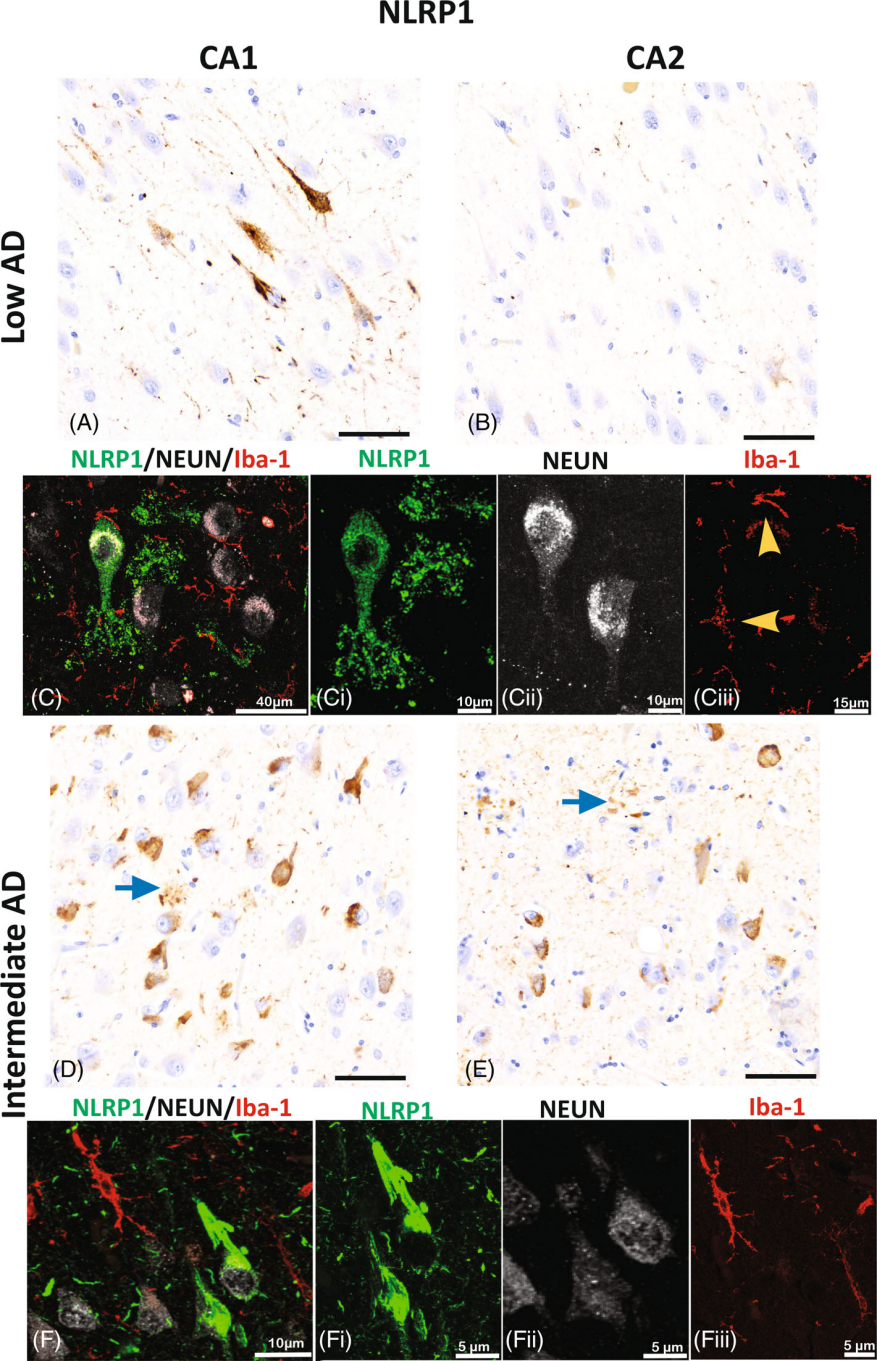
NLRP1 expression in hippocampal neurons. Immunoreactivity of NLRP1 in the CA1 (A and D) and CA2 (B and E) hippocampal regions in low AD (A–C) and in intermediate AD (D–F) cases. Expression of NLRP1 is present in neurons and apical dendrites in the CA1 region of low AD (A) more than in the CA2 (B) hippocampal region. In CA1, the cytoplasmic neuronal expression of NLRP1 (green, C and Ci) is confirmed by the confocal photomicrographs that show the expression in neurons (white; Cii), but not in microglia (yellow arrow heads; red; Ciii). In the intermediate AD, NLRP1 immunoreactivity is seen in numerous neurons and in parenchyma in the form of clusters (blue arrows) of the CA1 (D) and CA2 (E). The photomicrographs capture the dense neuronal cytoplasmic expression seen in CA1 of the intermediate AD cases (F and Fi) which is not present in the microglia (red; Fiii). Alzheimer's disease (AD); NOD‐like receptor proteins (NLRP); Cornu Ammonis Field 2 (CA2); Cornu Ammonis Field 1 (CA1); images A, B, C, and E; scale bar = 60 μm.

After identifying NLRP3 morphological changes in the intermediate AD cases, we set to look for changes in NLRP1 expression in the CA1/CA2 boarder zone. The low AD cases displayed perinuclear neuronal expression of NLRP1 in a few neurons of CA1 (Figure 4C), but not in CA2 regions (Figure 4A,B). In contrast, in the intermediate AD cases, there were more neurons with perinuclear neuronal expression in the CA1 and CA2 hippocampal regions (Figure 4D,E), indicating differential expression of NLRP1 in neurons within the CA1 and CA2 regions of the hippocampus associated with AD pathology. In all the cases, NLRP1 was seen in the cytoplasm of the neurons identified with the neuronal marker, but this expression was not seen in the microglia cells (Figure 4C,F).

### 3.7 Differential expression of ASC in neurons and microglia is detected using antibodies raised against different epitopes of ASC

**FIGURE 5:**
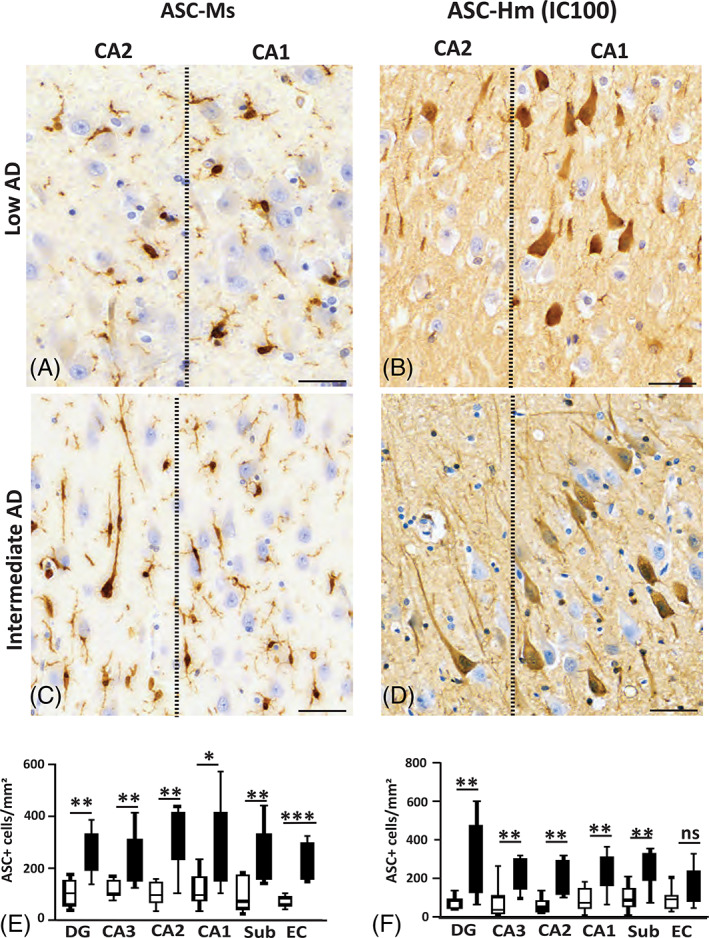
Differential expression of ASC in neurons and microglia. Mouse anti‐ASC (A, C, and E) cell type binding differs from IC100's in the hippocampal formation and entorhinal cortex (B, D, and F). Region‐specific significant changes in cell counts between intermediate cases and low AD cases (E and F). Different morphologies of microglia are present in the CA1‐CA2 boundary between low AD (A) and intermediate AD cases (C). Stereological analysis of cells stained with mouse anti‐ASC was significantly higher in intermediate AD cases in the DG, CA2, CA1 and sub regions, whereas IC100 stained mainly neurons in the DG, CA3, CA2 and CA1 hippocampal regions of intermediate cases (D) compared with low AD (B). Adaptor protein apoptosis‐associated speck‐like protein containing a caspase recruitment domain (ASC); dentate gyrus (DG); Cornu Ammonis Field 3 (CA3); Cornu Ammonis Field 2 (CA2); Cornu Ammonis Field 1 (CA1); subiculum (sub); entorhinal cortex (EC). Scale bar = 60 μm; * = *p* < 0.05; ** = *p* < 0.01; *** = *p* < 0.001; ns = *p* > 0.05.

Next, we assessed if brains with intermediate AD showed changes in expression of ASC in the hippocampal and entorhinal cortex regions. Mouse anti‐ASC was seen mainly on cells with microglial‐like morphology in the low AD group and intermediate AD brains (Figure 5A,C). In addition, there was a significant increase in the number of mouse anti‐ASC labeled microglia‐like cells in all the ROIs investigated. The EC (*p* = 0.0002; Intermediate AD 201 ± 87, *n* = 17; Low AD 86 ± 53, *n* = 17) and the subiculum (*p* = 0.01; Intermediate AD 214 ± 105, *n* = 17; Low AD 74 ± 65, *n* = 17). The DG (*p* = 0.001; Intermediate AD 236 ± 87, *n* = 17; Low AD 104 ± 53, *n* = 17), the CA3 (*p* = 0.002; Intermediate AD 214 ± 111, *n* = 17; Low AD 105 ± 46, *n* = 17), the CA2 (*p* = 0.002; Intermediate AD 301 ± 111, *n* = 17; Low AD 98 ± 46, *n* = 17), and the CA1 (*p* = 0.015; Intermediate AD 238 ± 162, *n* = 17; Low AD 99 ± 66, *n* = 17; Figure 5E) all demonstrated that microglial expression of ASC protein in upregulated in AD pathology.

The expression of ASC in human neurons during the progression of AD pathology is key to comprehending how AD progresses [43, 44]. Thus, we sought to address if ASC expression occurs at different rates across various regions of the hippocampus formation and the entorhinal cortex (Figure 5B,D). Hence, using IC100, an antibody specific to human ASC, we detected an increase in ASC expression mainly in neurons in the DG (*p* = 0.002; Intermediate AD 270 ± 189, *n* = 17; Low AD 49 ± 35, *n* = 17), the CA3 (*p* = 0.003; Intermediate AD 260 ± 84, *n* = 17; Low AD 39 ± 31, *n* = 17), the CA2 (*p* = 0.001; Intermediate AD 260 ± 93, *n* = 17; Low AD 51 ± 42, *n* = 17), the CA1 (*p* = 0.005; Intermediate AD 219 ± 93, *n* = 17; Low AD 72 ± 42, *n* = 17), and the subiculum (*p* = 0.00; Intermediate AD 267 ± 93, *n* = 17; Low AD 86 ± 42, *n* = 17). Interestingly, there were no significant differences in the number of neurons that were detected with IC100 in the EC, the region that commonly present in the early stages of AD pathology (*p* > 0.05; Figure 5F). This could either exemplify a temporal response or that antibodies specific to human ASC identify a response that is specific to human neurons.

### 3.8 Caspase‐1 expression is seen in the parenchyma of low AD and intermediate AD cases

**FIGURE 6:**
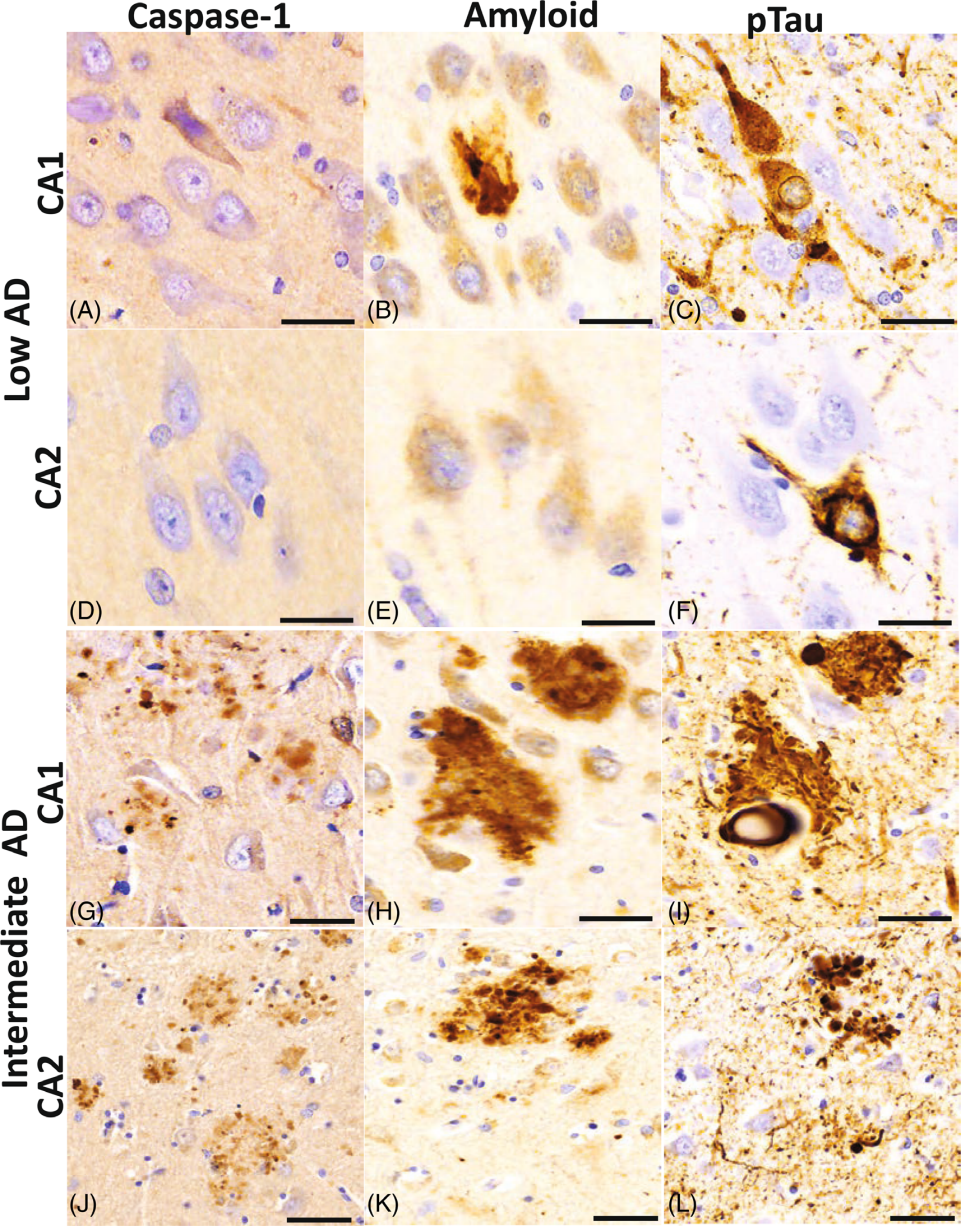
Caspase‐1 protein expression in the CA1 and CA2 hippocampal regions. Distribution of caspase‐1 adjacent to neurons and in the tissue parenchyma in clusters that resemble amyloid deposits. The CA1 (A) region of the low AD cases shows a sparse cluster of caspase‐1 in the vicinity of an Aβ plaque (B) and a neurofibrillary tangle and neuritic threads (C). In CA2, a region less affected by low AD neuropathological changes, there is no caspase‐1 immunoreactivity defined (D) nor Aβ positive plaques (E) but there is an occasional neurofibrillary tangle present (F). Intermediate AD cases have denser cluster expression of caspase 1 in both the CA1 (G) and CA2 (J) regions in areas where moderate densities of Aβ plaques (H and K) and neurofibrillary tangles and threads are noted (J and L). Beta‐amyloid (Aβ); hyperphosphorylated (ptau); Cornu Ammonis Field 2 (CA2); Cornu Ammonis Field 1 (CA1); Alzheimer's disease (AD); scale bar = 30 μm.

The most substantial pathological changes between intermediate AD and control cases are evident between the CA1 and the CA2 hippocampal regions [38]; therefore, we focused on these regions to further investigate the expression of caspase‐1. Caspase‐1 expression was present in the tissue parenchyma in areas where diffuse amyloid plaque‐like formation and pTau neurofibrillary tangles were seen in the CA1 region of both the low AD (Figure 6A–C) and intermediate AD (Figure 6G–I) cases. Importantly, the CA2 region showed more prominent caspase‐1 expression, amyloid plaque formations, and pTau neurofibrillary tangles in the intermediate AD cases (Figure 6J–L) compared to low AD (Figure 6D–F), suggesting an increase in caspase‐1 activity, and as a result the inflammasome, in the hippocampus of patients with intermediate AD.

## 4 DISCUSSION

In this study, using a panel of human‐specific inflammasome antibodies, we compared the expression of the inflammasome signaling proteins NLRP1, NLPR3, ASC, and caspase‐1 in the brain of donors with cases presenting intermediate AD and low AD pathology. Our findings indicate that in early AD pathology, before cell and hippocampal volume loss occurs, neurons and microglia exhibit increased inflammasome protein expression. NLRP1 was primarily expressed in neurons, whereas NLRP3 was present in microglia. In addition, caspase‐1 was present in the tissue parenchyma of the hippocampus. Importantly, IC100 identified increased ASC expression in neurons in the early stages of AD, whereas a commercially available antibody directed toward a different domain of the ASC protein (the CARD domain), primarily labeled microglia. To our knowledge, this is the first demonstration of ASC expression in distinct cell populations during the early stages of AD and highlights the importance of the inflammasome in the early stage of AD pathology.

Recent evidence has accumulated that inflammasome‐induced cytokines and inflammasome signaling proteins released from activated microglia interact with AD‐associated proteins and exacerbate AD pathological progression and cellular damage. The expression of NLRP3 in microglia of low and intermediate AD cases has been previously reported [45] NLRP3 may contribute to chronic neuroinflammation via the production of IL‐1β, resulting in reduced clearance of Aβ plaques [7, 46, 47, 48]. In the intermediate AD cases, NLRP3 expression was seen in microglia adjacent to neurons or was present in a clustered formation. It is possible that the cluster distribution of NLRP3 configuration may be mediated by TANK‐binding kinase 1 (TBK1) that interacts with tau proteins [49]. Moreover, we demonstrated that NLRP1 is expressed in the cytoplasm of hippocampal neurons, and is upregulated in cases with intermediate AD. This observation is consistent with our previous studies that show NLRP1 is present in motor neurons in the ventral horn of the human spinal cord and is upregulated after spinal cord injury [8], traumatic brain injury [18], stroke [50], and the aging brain [23, 51]. In addition, Saresella et al. [48], Yap et al. [44], and Kaushal et al. [52] reported that NLRP1 is primarily expressed by pyramidal neurons of the hippocampus and is activated by aggregated Aβ. A recent study found that NLRP1 knockout in mouse models of AD resulted in reduced Aβ plaque load, normalized hippocampal dendritic spines, and resulted in improved spatial and episodic memory testing performance [53]. Taken together, our findings indicate that the NLRP1 inflammasome plays a role in the neurodegenerative process in neurons, in addition to NLRP3 inflammasome upregulation in microglia. However, it is unclear whether other inflammasomes harbored in various CNS cell types also contribute to the heightened inflammatory response in AD and whether tau, Aβ or ASC specks released from inflammasome‐activated cells trigger cell death processes such as pyroptosis, which may contribute to the demise of hippocampal cells in early stages of AD.

The ages of the cohort examined in this study were similar in the intermediate AD and low AD groups, but cognitive impairment was higher in the intermediate AD group. We cannot exclude the possibility that AD would have progressed in some low AD cases if donors had lived longer. Nevertheless, our data suggest that changes in the inflammasome protein expression in neurons and microglia accompany the neurodegeneration of AD in the central nervous system of aging individuals.

The hippocampal formation cortical band did not differ in thickness between low AD and intermediate AD, indicating that neuronal death and atrophy were not evident in these cases. Two other studies examined hippocampal thickness and neuronal numbers in AD cases staged at different Braak stages V–VI [41, 42] and reported hippocampal neuronal loss at Braak stages V–VI, thus supporting our findings. In that study, the authors evaluated neuropathological changes between the low AD and the intermediate groups in all of the hippocampal CA regions [9, 38] and found that the number of Aβ plaques clusters did not significantly differ between the low and intermediate groups. However, here we found that the morphology of the plaques in the intermediate group was more dense, which resembled neuritic plaques, rather than diffuse plaques as those seen in low AD cases. In contrast, in the intermediate group, we saw a significant increase in the number of neurons labeled with pTau in the subiculum, CA1, CA2, and DG regions, consistent with previous studies on tau pathology in postmortem AD cases [37, 38, 54, 55].

Protein aggregation has been connected to more than 30 proteinopathies, including many neurodegenerative diseases such as AD in which both Aβ and tau aggregates are present [56, 57]. Recent evidence indicates that heterotypic interactions of aggregated proteins occur in a wide range of amyloid processes and that these interactions modify fundamental aspects of amyloid aggregation including seeding, aggregation rates, and toxicity [58]. In this study, we examined the expression of three protein aggregates, ASC, Aβ, and p‐tau. The expression of all three aggregated proteins was higher in intermediate AD compared to low AD, indicating increased deposition associated with disease progression. In AD pathology, it has been shown that ASC specks cross‐seed with Aβ_1–42_, in the extracellular space, which boosts Aβ_1–42_ toxicity in microglia [11, 59], suggesting that disease‐related protein aggregation may modulate the morphology or aggregation rate of amyloidogenic proteins [58]. In support of this idea is the observation that excessive inflammasome stimulation in microglia and neurons induces the oligomerization of ASC to form the inflammasome complex, and that this response is exacerbated by tauopathies [16]. Our result that ASC specks are present in the brain of AD patients suggests that immunotherapies that target protein aggregates may be promising targets for the treatment of neurodegenerative diseases.

The inflammasome adaptor ASC has different isoforms in addition to the canonical ASC. These other isoforms are referred to as ASCb, ASCc, and ASCd, and they differ in amino acid composition [60]. ASC and ASCb are very similar, and both contain PYD and CARD domains. However, they differ in the length of the interdomain linker. ASCc contains a CARD domain and a fragment of the PYD, whereas ASCd has a partial PYD and no CARD domain. Full‐length ASC and ASCb colocalize with the sensor NLRP‐3 and procaspase‐1 [60] and activate the inflammasome [61]. However, ASCb does not form typical ASC specks but rather forms filamentous aggregates of ASC resulting in lower levels of inflammasome activation as determined by IL‐1β release [60]. ASC oligomerizes faster and is capable of assembling into oligomers of more uniform size, compared with ASCb [62]. In contrast, ASCc only colocalizes with caspase‐1, and it may inhibit the inflammasome in that it diminishes the release of IL‐1β in the presence of ASC. ASCd, whose function is yet to be determined, does not colocalize with NLRP3 and cannot generate mature IL‐1β [60]. It is possible that distinct combinations of ASC splice variants may be expressed in cells that potentially affect inflammasome activity at different stages of the inflammatory response [60]. Our results show that antibodies raised against two different epitopes of ASC (CARD vs. PYD) differentially identify neurons and microglia in the early stages of AD, thus suggesting that ASC may either be present in different conformation states within the different population of CNS cells or that there are different ratios of distinct ASC isoforms expressed in microglia and neurons that are differentially recognized by the two antibodies. Moreover, the expression of ASC in the early stages of AD pathology is consistent with our previous work showing that ASC is elevated in the blood of patients with mild cognitive impairment and that ASC is a reliable biomarker of AD [63]. Accordingly, ASC and IL‐18 were both significantly elevated in the serum of MCI patients when compared to controls, whereas ASC protein levels were also higher in the serum of MCI patients when compared to AD patients [63]. Furthermore, future studies are needed to understand the expression of different ratios of activating and inhibitory isoforms of ASC in cells and how they might promote inflammation at the early stages of infections and tissue damage.

Previous studies have reported inflammasome signaling proteins in neurons and microglia in brain of donors with AD [46, 47, 48, 64], while others studies show that progressive inflammatory signals occur in activated astrocytes [65, 66]. In our investigations, we show that in AD, the progression of phosphorylated tau is in concert with an increase of NLRP3 in microglial and NLRP1 in neurons. In addition, we show that caspase‐1 is present in regions where parenchymal beta‐amyloid plaques are present. Previous studies have shown that NLRP3 inflammasome activation is prevalent in AD mouse models and in human postmortem brains [7, 16, 46, 47] and suggest that inhibition of this sensor protein may be a therapeutic target for AD. However, NLRP1 may be upregulated in neurons [44, 52] as neuronal degeneration progresses in AD. It is not known if inhibition of one sensor, such as NLRP3 or NLRP1, could prevent the oligomerization of ASC. In addition, the inhibition of caspase‐1 has been shown to inhibit cognitive decline in mouse models of AD by reducing the production of active caspase‐6 [67].

In this study, we expand that knowledge by comparing the cell‐specific staining characteristics of two ASC antibodies, one against the CARD of ASC and the other, IC100, against the PYD, and a panel of commercially available antibodies to identify Ab, pTau, and the inflammasome proteins NLRP1, NLRP3 and caspase‐1. Compared to a commercially available mouse ASC antibody, IC100 identified neurons in the early stages of neurodegeneration in intermediate stages of AD. IC100 expression patterns were consistent with Aβ and pTau staining in postmortem AD brains, emphasizing a spatial and temporal relationship of this sensor molecule in the pathogenesis of AD neuropathology. The reported differential patterns of IC100 neuronal immunostaining in human AD specimens without evidence for cell loss may emphasize the importance of targeting this unique ASC configurational state for future molecular imaging and therapeutic approaches to reduce neuronal vulnerability and the subsequent release of ASC specks leading to continued disease progression.

Several surrogate markers are under development to assess initial signs of disease emergence, including molecular neuroimaging, blood or CSF biomarkers, and sensitive indicators of cognitive disturbances [68, 69, 70]. In addition, numerous studies and potential therapeutic interventions are currently being investigated to prevent, reduce, or reverse the effects of AD pathology. The inflammasome pathway offers attractive therapeutic targets for the reduction of damaging inflammatory secondary injury cascades. IC100 is a humanized monoclonal antibody against the adaptor protein ASC [71] that has therapeutic benefits in treating several experimental models for neurodegeneration [22], injury [72, 73], and aging [23]. In this study, IC100 immunoreactivity demonstrated cell‐specific labeling patterns based on regional specificity and AD pathological severity in contrast to the commercially available mouse anti‐ASC antibody that was primarily immunoreactive for cells with microglial‐like morphology.

## 5 FINAL SUMMATION

In conclusion, our findings in the brain of donors with low and intermediate AD pathology indicate that the NLRP1 inflammasome is mainly present in neurons, whereas the NLRP3 inflammasome is mainly present in microglia. Moreover, ASC is present in neurons and microglia. However, the antibody against the CARD of ASC mainly detected ASC in microglia; whereas IC100, which recognizes the PYD of ASC mainly detected neurons. Thus, our data suggest that IC100 identifies neurons in the early stages of neurodegeneration in intermediate stages of AD and that ASC expression is consistent with the levels of Aβ and pTau in postmortem AD brains.

## CONFLICTS OF INTEREST

JPdRV, HMB, RWK, and WDD are co‐founders and managing members of InflamaCORE, LLC and have licensed patents on inflammasome proteins as biomarkers of injury and disease as well as on targeting inflammasome proteins for therapeutic purposes. JPdRV, HMB, RWK, and WDD are Scientific Advisory Board Members of ZyVersa Therapeutics. NHJ and RTV declare no conflicts of interest.

## ETHICS STATEMENT

Research study ethics was obtained from the Human Subjects Research Office, University of Miami, Miami, Florida (IRB ethics number, 19920348 (CR00012340); Brain Endowment Bank).

## Supporting information


**FIGURE S1.** Protein expression in tonsil and skin tissue

**FIGURE S2.** Alzheimer pathology seen in hematoxylin and eosin staining in two donors with intermediate Alzheimer's disease neuropathological changes

## Data Availability

The data that support the findings of this study are available on request from the corresponding author. The data are not publicly available due to privacy or ethical restrictions.
