# Supplementary material for: Identification of inflammasome signaling proteins in neurons and microglia in early and intermediate stages of Alzheimer's disease
Source: Brain Pathol. 2022 Dec 29;33(4):e13142. doi: 10.1111/bpa.13142 (PMC10307529; doi:10.1111/bpa.13142)
Supplement: Supplementary file 1 — FIGURE S1. Protein expression in tonsil and skin tissue [file BPA-33-e13142-s002.pdf]

Supplementary Data

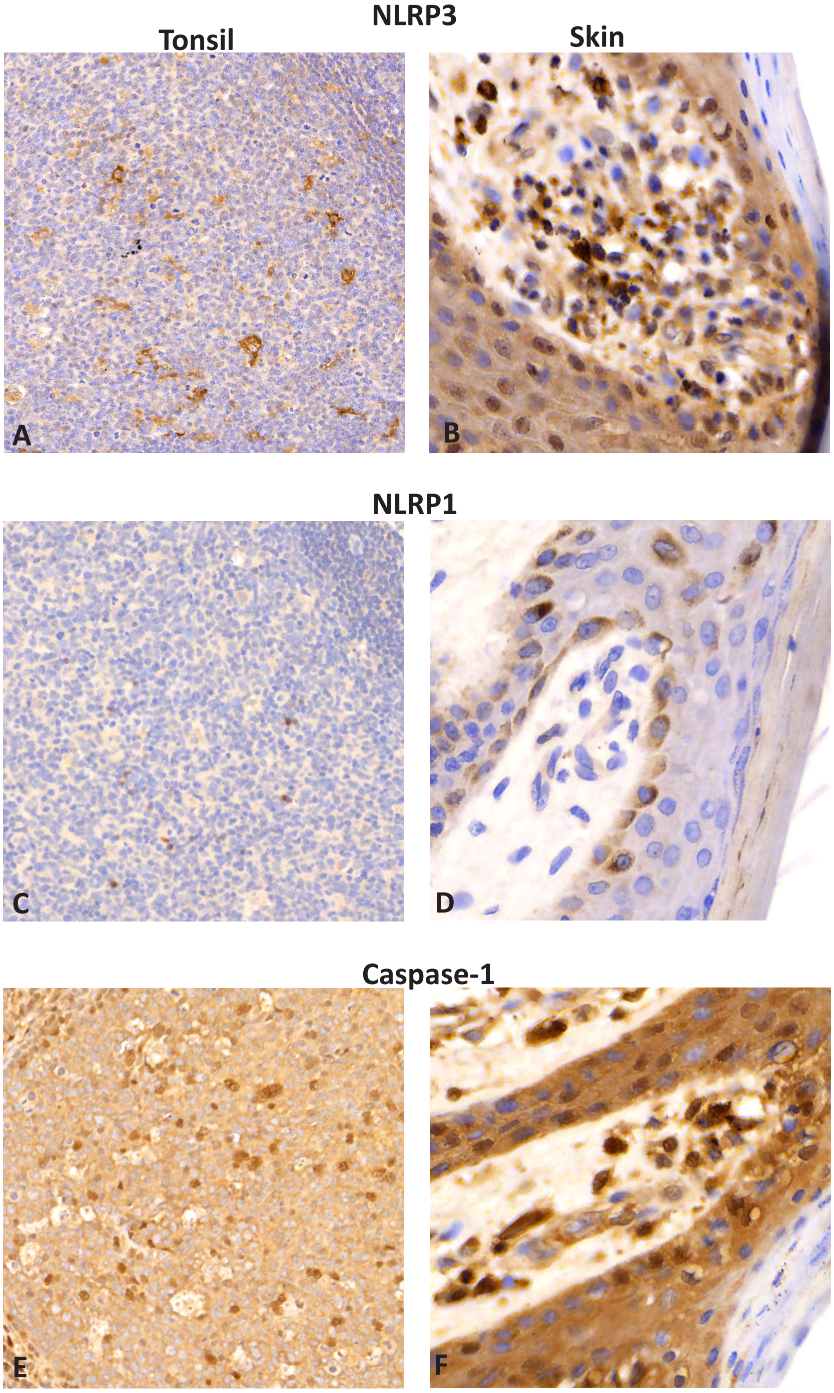

Supplementary Data 1. Protein expression in tonsil and skin tissue. Protein expression of NLRP3, NLRP1 and caspase-1 on tonsil (A, C and E) and skin (B,D and F). In images A and B, the NLRP3 expression is seen germinal center in tonsil (A) and the cells of the epidermis of the skin (B). The NLRP1 expression is need in lower levels of the germinal center in tonsil (C) and is seen mainly in the Langerhans cells of skin (D). Moderate expression of caspase-1 immunopositivity was seen in the tonsil (E) and in skin (F). NOD-like receptor proteins (NLRP).
