# Supplementary material for: Identification of inflammasome signaling proteins in neurons and microglia in early and intermediate stages of Alzheimer's disease
Source: Brain Pathol. 2022 Dec 29;33(4):e13142. doi: 10.1111/bpa.13142 (PMC10307529; doi:10.1111/bpa.13142)
Supplement: Supplementary file 2 — FIGURE S2. Alzheimer pathology seen in hematoxylin and eosin staining in two donors with intermediate Alzheimer's disease neuropathological changes [file BPA-33-e13142-s001.pdf]

Supplementary Data 2

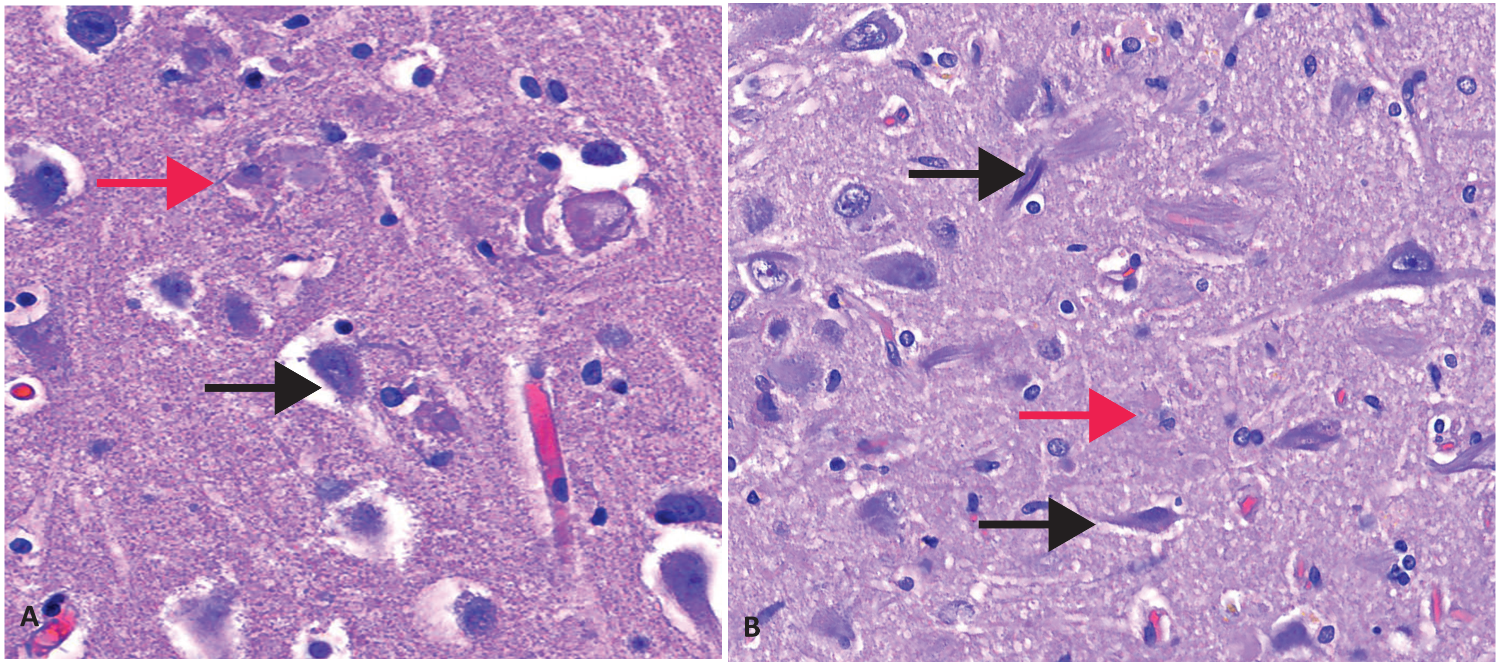

Supplementary Data Figure 2. Alzheimer pathology seen in Hematoxlyin and Eosin staining in two donors with intermediate Alzheimer's disease neuropathological changes. In the Intermediate AD donor #16, the red arrows are pointing to amyloid plaque formation and the black arrow is pointing to a neurofibillary tangle seen in the CA 2 region of the hippocampus (image A). The CA1 hippocampal region is shown in image B from intermediate AD donor #3. The black arrows are pointing to neurofibillary tangles and the red arrow is pointing to a small plaque formation.
